# A Clinical–Radiomics Nomogram for the Preoperative Prediction of Aggressive Micropapillary and a Solid Pattern in Lung Adenocarcinoma

**DOI:** 10.3390/curroncol32060323

**Published:** 2025-05-30

**Authors:** Xiangyu Xie, Lei Chen, Kun Li, Liang Shi, Lei Zhang, Liang Zheng

**Affiliations:** Department of Thoracic Surgery, The First People’s Hospital of Changzhou and The Third Affiliated Hospital of Soochow University, Changzhou 213000, China; 20235235063@stu.suda.edu.cn (X.X.); 20225235052@stu.suda.edu.cn (L.C.); 20245235071@stu.suda.edu.cn (K.L.); suda20010131@163.com (L.S.); suda5235052@163.com (L.Z.)

**Keywords:** lung adenocarcinoma, radiomics, clinical independent factors, micropapillary and solid patterns

## Abstract

**Background:** A micropapillary pattern (MP) and solid pattern (SP) in lung adenocarcinoma (LUAD), a major subtype of non-small-cell lung cancer (NSCLC), are associated with a poor prognosis and necessitate accurate preoperative identification. This study aimed to develop and validate a predictive model combining clinical and radiomics features for differentiating a high-risk MP/SP in LUAD. **Methods:** This retrospective study analyzed 180 surgically confirmed NSCLC patients (Stages I–IIIA), randomly divided into training (70%, n = 126) and validation (30%, n = 54) cohorts. Three prediction models were constructed: (1) a clinical model based on independent clinical and CT morphological features (e.g., nodule size, lobulation, spiculation, pleural indentation, and vascular abnormalities), (2) a radiomics model utilizing LASSO-selected features extracted using 3D Slicer, and (3) a comprehensive model integrating both clinical and radiomics data. **Results:** The clinical model yielded AUCs of 0.7975 (training) and 0.8462 (validation). The radiomics model showed superior performance with AUCs of 0.8896 and 0.8901, respectively. The comprehensive model achieved the highest diagnostic accuracy, with training and validation AUCs of 0.9186 and 0.9396, respectively (DeLong test, *p* < 0.05). Decision curve analysis demonstrated the enhanced clinical utility of the combined approach. **Conclusions:** Integrating clinical and radiomics features significantly improves the preoperative identification of aggressive NSCLC patterns. The comprehensive model offers a promising tool for guiding surgical and adjuvant therapy decisions.

## 1. Introduction

### Background

Lung cancer remains as one of the most prevalent malignancies worldwide and is the leading cause of cancer-related mortality, accounting for approximately 2 million new cases and 1.76 million deaths annually [1]. The complexity of early-stage lung nodules and tumor heterogeneity, coupled with diverse contributing factors, often limits treatment strategies and contributes to poor prognostic outcomes [2]. Due to the nonspecific clinical presentations and limited sensitivity of existing diagnostic tools, early detection remains challenging, resulting in approximately 75% of patients being diagnosed in the advanced stages [3]. Consequently, the five-year survival rate for lung cancer remains below 18% [4].

Lung cancer is broadly categorized into small-cell lung cancer (SCLC) and non-small-cell lung cancer (NSCLC), with the latter comprising 85–90% of cases. NSCLC encompasses various histological patterns, including lung adenocarcinoma (LUAD), squamous cell carcinoma, and large-cell carcinoma [5]. LUAD itself exhibits a spectrum of histological patterns, such as lepidic, acinar, papillary, micropapillary, and solid patterns. The 2021 World Health Organization (WHO) classification recommends quantifying each component in 5% increments, with the predominant pattern guiding the prognosis and management decisions [6,7].

Mounting evidence suggests that MP and SP subtypes are associated with more aggressive biological behavior and worse clinical outcomes. A meta-analysis involving 19,502 LUAD patients across 48 studies confirmed that the presence of MP and SP components correlates with increased recurrence, lymph node metastasis, and significantly reduced overall survival [8,9]. These patterns not only influence the recurrence risk post-lobectomy but also play a critical role in determining the need for adjuvant chemotherapy [10,11,12].

Surgical resection remains the cornerstone of treatment for early-stage LUAD, with lobectomy or sublobar resection being commonly employed to achieve oncological clearance while preserving lung function [13,14]. The increasing use of imaging technologies such as digital radiography, CT, MRI, PET/CT, and the adoption of low-dose CT (LDCT) screening programs have improved the early detection of pulmonary nodules. However, LDCT often yields a high false-positive rate and lacks histologic specificity, underscoring the need for enhanced preoperative risk stratification tools [15,16]. Histopathologic evaluation remains the gold standard for LUAD subtype classification; however, it relies on invasive procedures such as percutaneous biopsy or surgical resection, which may be constrained by tumor size, anatomical location, or the patient’s clinical condition [17]. Moreover, tissue samples obtained preoperatively may not adequately capture the full spectrum of tumor heterogeneity, leading to the potential underestimation of aggressive subtypes such as micropapillary and solid components. These limitations have stimulated growing interest in non-invasive approaches that can preoperatively predict histologic patterns and inform personalized treatment strategies.

Radiomics, an emerging field that enables high-throughput extraction of quantitative imaging features from medical scans, offers a promising solution. These features reflect the underlying tumor phenotype, architecture, and microenvironment, and may serve as imaging biomarkers associated with tumor aggressiveness and specific histologic subtypes such as MP and SP [17,18,19,20]. However, the clinical translation of radiomics models remains challenged by issues such as feature reproducibility, imaging protocol heterogeneity, and a lack of external validation [21,22].

Therefore, the primary aim of this study is to develop and validate a radiomics-based predictive model, integrating both imaging features and clinical variables, for the preoperative identification of MP and SP components in lung adenocarcinoma. By offering a non-invasive tool to stratify the histologic risk before surgery, the proposed model may support more accurate treatment planning, guide the need for adjuvant therapy, and ultimately improve patient outcomes.

## 2. Materials and Methods

### 2.1. Study Design and Population

**Figure 1:**
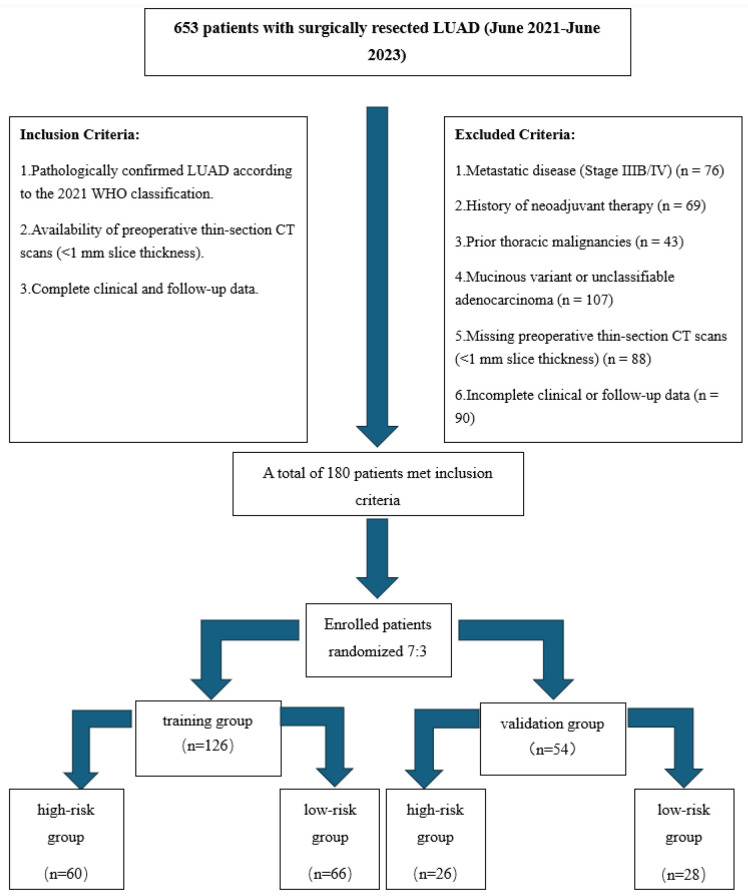
Flowchart of patient screening and grouping. Clinical data collection: demographic (age, sex), anthropometric (height, weight, BMI), and clinical characteristics (e.g., smoking history) were extracted from electronic medical records by two independent investigators.

This retrospective study was approved by the institutional review board and adhered to the STROBE guidelines for observational research. A total of 180 consecutive patients with surgically resected LUAD (Stages I–IIIA) between June 2021 and June 2023 were enrolled. The flowchart of the screening and grouping is shown in Figure 1.

### 2.2. CT Acquisition and Radiomics Standardization

To ensure the reproducibility and standardization of the radiomics features, all CT scans were performed using a 640-slice multidetector CT scanner (Aquilion ONE, Canon Medical Systems, Otawara, Tochigi Prefecture, Japan) under a uniform acquisition protocol. The technical parameters were as follows:

1. Tube voltage: 120 kVp with automatic tube current modulation (SD 12–15).
2. Rotation time: 0.35 s per rotation.
3. Reconstruction kernel: FC81 (sharp), 1 mm isotropic voxels.
4. Radiation dose: median CTDIvol of 3.2 mGy (range: 2.8–3.6 mGy); DLP: 110–150 mGy·cm.
5. Contrast-enhanced CT was obtained during the venous phase (60–70 s post-injection) using Meglumine Diatrizoate (65% *w*/*v*, 306 mgI/mL). A dual-syringe injector was used to deliver 1.5–2.0 mL/kg (maximum 100 mL) of contrast at 2.5–3.0 mL/s, followed by a 30 mL saline flush. Patients fasted for ≥4 h before scanning, and renal function was verified within 72 h (eGFR > 45 mL/min/1.73 m^2^).

#### 2.2.1. Image Analysis

Two thoracic radiologists (each with >5 years of experience), blinded to patient outcomes, evaluated the following CT features:

Nodule size (maximum axial diameter) and type (pure ground-glass opacity, sub-solid opacity with a consolidation tumor ratio (CTR) less than 50%, sub-solid opacity with a CTR greater than 50%, and a pure solid nodule).

Morphological signs: lobulation, spiculation, vacuole sign, and bronchial abnormality sign.

Pleural and vascular relationships (e.g., pleural indentation, vascular abnormality sign) [23,24,25,26].

#### 2.2.2. Pathological Evaluation

Two pulmonary pathologists reviewed resection specimens based on the 2021 WHO criteria. The dominant histological pattern was determined, with discrepancies resolved by consensus.

#### 2.2.3. Radiomics Workflow

Radiomics image processing followed the Image Biomarker Standardization Initiative (IBSI) guidelines to ensure feature reproducibility. Preprocessing and feature extraction steps included the following:

1. Gray-level discretization using a fixed bin width of 25 HU within the intensity range of −1000 to 400 HU.
2. Wavelet decomposition using a 3-level Haar transform (yielding 8 wavelet subbands).
3. ROI segmentation was manually performed using 3D Slicer (v5.2.2) in three orthogonal planes.
4. Feature extraction was conducted using PyRadiomics (v3.0) or the SlicerRadiomics extension, compliant with IBSI standards.
5. The extracted features comprised first-order statistics, 2D/3D shape descriptors, and second-order texture features from the GLCM, GLRLM, GLSZM, and NGTDM matrices.

To ensure measurement robustness, intra- and inter-observer reproducibility was assessed using the intraclass correlation coefficient (ICC) on a 20% random sample of patients, with features achieving an ICC > 0.8 being retained for further modeling.

### 2.3. Statistical Analysis

#### 2.3.1. Clinical Model Development

1. Continuous variables: normality tested via the Shapiro–Wilk test, *t*-test, or Mann–Whitney U test, applied accordingly.
2. Categorical variables: χ^2^ test or Fisher’s exact test.
3. Variables significant in the univariate analysis were entered into multivariate logistic regression with forward selection.

#### 2.3.2. Radiomics Model Development

1. Prior to feature selection, all radiomics features were standardized using Z-score normalization. Subsequently, the least absolute shrinkage and selection operator (LASSO) regression with 10-fold cross-validation was performed to select the most predictive features.
2. The radiomics score (RadScore) was calculated as a linear combination of the selected features weighted by their LASSO coefficients, using the original (non-normalized) feature values.

Comprehensive Model:

1. Combined clinical and radiomics predictors were incorporated into a multivariate logistic regression model (ComScore).
2. A nomogram was constructed to visualize the model.

**Figure 2:**
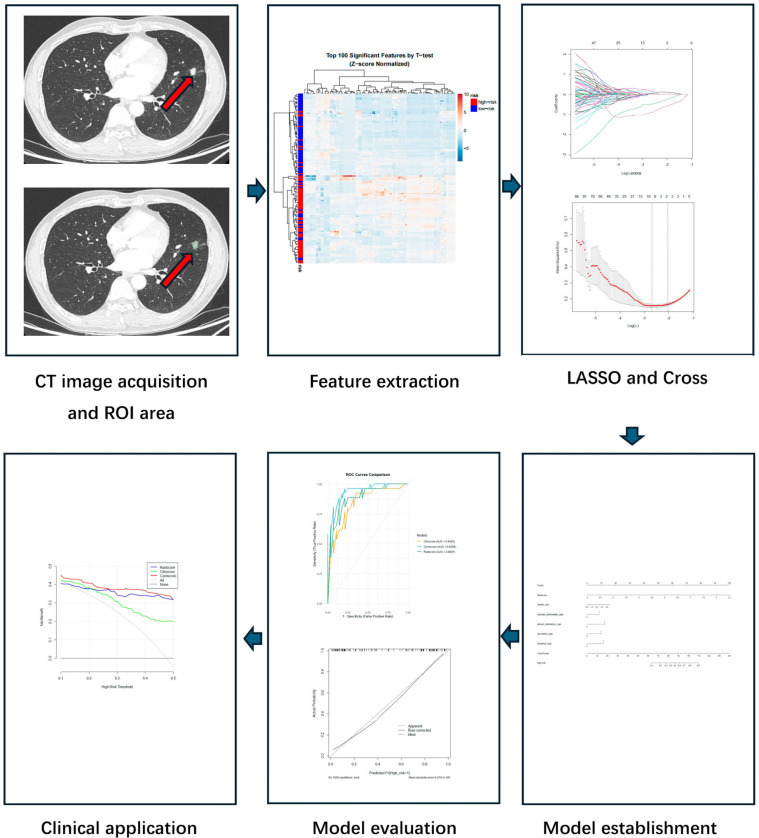
Workflow of radiomics and comprehensive model development. The pipeline consisted of the following steps: (1) **CT image acquisition and ROI delineation**, where the lesion region was manually segmented on axial images; (2) **radiomics feature extraction**, using IBSI-compliant methods to quantify tumor characteristics; (3) **feature selection**, performed via the LASSO regression with 10-fold cross-validation; (4) **model establishment**, where selected features were integrated into a logistic regression model and visualized as a nomogram; (5) **model evaluation**, using ROC analysis and calibration curves to assess performance; (6) **clinical application**: decision curve analysis (DCA) was used to evaluate the net benefit and potential clinical application of the model.

The detailed workflow of radiomics analysis and comprehensive model construction is illustrated in Figure 2.

### 2.4. Model Validation and Comparison

1. **Discrimination**: Receiver operating characteristic (ROC) curve analysis was performed for each model in both the training and validation cohorts. The area under the curve (AUC) and corresponding 95% confidence intervals (CIs) were calculated to quantify the discriminative ability of each model in distinguishing between patients with and without a pneumothorax.
2. **Calibration**: Calibration performance was assessed using calibration curves based on 1000 bootstrap resamples. This evaluation was conducted to determine the agreement between the predicted probabilities and the observed outcomes, ensuring the model’s reliability in clinical settings.
3. **Clinical Utility**: DCA was employed to evaluate the net clinical benefit of each model across a range of threshold probabilities. This method reflects the potential utility of the models in real-world decision-making by quantifying the trade-off between true positives and false positives at varying decision thresholds.
4. **Comparative Performance**: To assess whether differences in discrimination among models were statistically significant, DeLong’s test was applied to compare the AUC values between pairs of models in both cohorts.
5. **Threshold Selection and Diagnostic Performance**: The optimal classification threshold for each model was determined by maximizing the Youden index, which identifies the point that optimizes the trade-off between sensitivity and specificity. At the Youden-derived threshold, additional diagnostic metrics—including sensitivity, specificity, the positive predictive value (PPV), the negative predictive value (NPV), accuracy, and the F1 score—were computed to provide a comprehensive evaluation of each model’s diagnostic performance and clinical applicability. All statistical analyses were performed using R software (version 4.2.2), with significance defined as a two-sided *p*-value < 0.05.

## 3. Results

### 3.1. Clinical Model Development

Univariate analysis in the training cohort revealed that nodule size (*p* = 0.010), lobulation (*p* = 0.009), spiculation (*p* = 0.005), vacuole sign (*p* = 0.017), pleural indentation (*p* < 0.01), and vascular abnormality (*p* = 0.026) were significantly associated with a high-risk pathological pattern. Variables such as age, gender, BMI, nodule type and smoking history did not show statistical significance (*p* > 0.05).

In the validation cohort, these associations remained consistent, with significant differences observed in nodule size (*p* < 0.001), lobulation (*p* = 0.034), spiculation (*p* = 0.013), vacuole sign (*p* = 0.012), pleural indentation (*p* = 0.028), and vascular abnormality (*p* = 0.002). Interestingly, age reached significance in the validation set (*p* = 0.005) but not in the training set. The data are presented in Table 1.

Multivariate logistic regression identified five independent predictors: nodule size, lobulation, spiculation, pleural indentation, and vascular abnormality. The resulting clinical model (ClinScore) was formulated as follows:

$$\begin{array}{cc} ClinScore=- & 4.208+0.428\times (nodule\;size)\\ & +1.140\times (vascular\;abnormality)\\ & +1.556\times (pleural\;indentation)\\ & +1.114\times (spiculation)+1.339\times (lobulation) \end{array}$$

### 3.2. Radiomics Model Development

**Figure 3:**
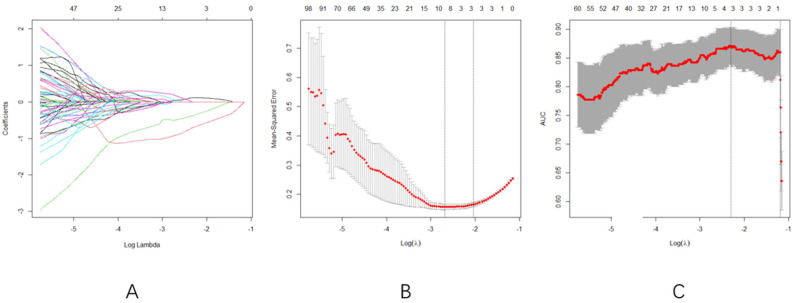
(**A**)**:** Radiomics features and their corresponding eigenvalues under different Log lambda values, (**B**): the value of Log lambda and the number of radiomics features are fixed according to the cross-validation results, and (**C**): AUC trajectory. Consistency with Figure 3B confirms robust feature selection within the same log(λ) range.

The Feature Selection Process Demonstrated the following:

1. Optimal Regularization: At log(λ) = −2.1 (Figure 3A), the model achieved equilibrium between feature sparsity (3 features retained) and predictive performance.
2. Cross-validation Consistency: Both MSE and AUC plateaued in the log(λ) range of −3 to −2 (Figure 3B,C), confirming the selection stability
3. LASSO regression with 10-fold cross-validation selected three radiomics features: original-firstorder-median, original-firstorder-skewness, and original-firstorder-clustershade.

The RadScore was computed as follows:

RadScore=−0.2994+0.0004×Median−0.5829×Skewness−0.00007×ClusterShade

### 3.3. Comprehensive Model Development

A multivariate logistic model was developed combining the clinical predictors and the RadScore. The final formula for the comprehensive model (ComScore) was as follows:

$$\begin{array}{cc} ComScore= & 0.331\times (nodule\;size)+0.812\times (vascular\;abnormality)\\ & +1.177\times (pleural\;indentation)+0.940\times (spiculation)\\ & +1.090\times (lobulation)+1.737\times RadScore-3.218 \end{array}$$

**Figure 4:**
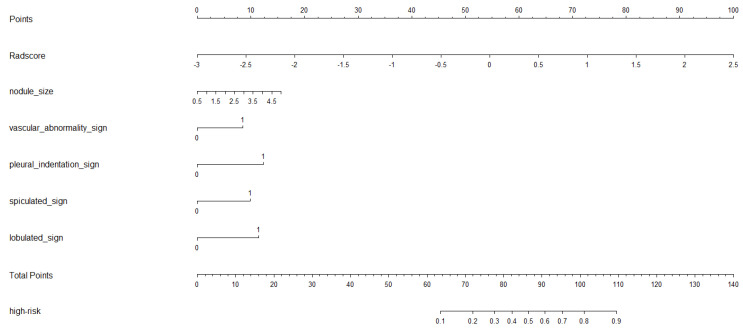
The nomogram of comprehensive model.

A nomogram representing this comprehensive model is shown in Figure 4. The ROC curves for the ComScore were generated using data from both the training and validation groups.

### 3.4. Model Comparison and Validation

**Figure 5:**
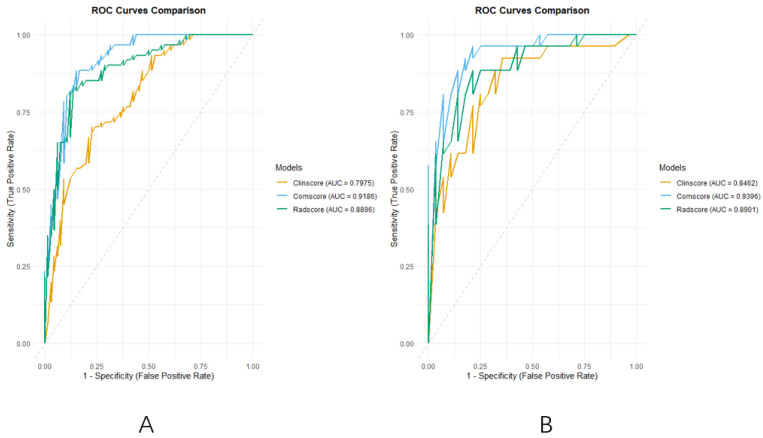
(**A**): The ROC curves of the three models of the training group; (**B**): the ROC curves of the three models of validation group.

Analysis of the ROC curves (Figure 5A,B) further demonstrated that the radiomics and comprehensive models provided superior diagnostic efficiency compared to the clinical model.

The DeLong test confirmed that the comprehensive model significantly outperformed the clinical model in both the training (Z = −3.586, *p* = 0.0003) and validation cohorts (Z = −2.233, *p* = 0.0255). In the training cohort, the radiomics model also outperformed the clinical model (Z = −2.053, *p* = 0.0401), though its advantage was not statistically significant in the validation cohort (Z = −0.658, *p* = 0.5107).

**Figure 6:**
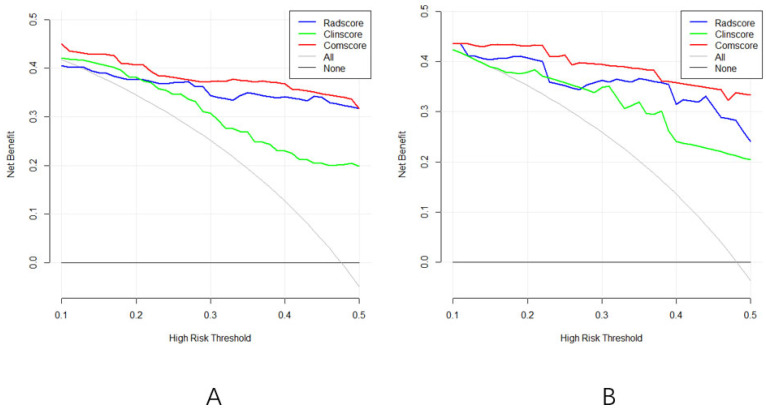
(**A**): The DCA curve of the three models of the training group; (**B**): the DCA curves of the three models of the validation group.

The clinical utility of the prediction models was assessed through DCA, which evaluates the net benefit of taking clinical action versus no action at various probability thresholds. The DCA curves for the three diagnostic models in both the training and validation sets (Figure 6A,B) revealed a net survival benefit for all models. Notably, the radiomics and comprehensive models exhibited higher net survival benefits compared to the clinical model.

In both the training and validation groups, we systematically evaluated six performance metrics across the three models: sensitivity, specificity, the PPV, the NPV, accuracy, and the F1 score. In the training set, the comprehensive model demonstrated the best performance, with a sensitivity of 88.33%, an NPV of 88.89%, accuracy of 86.51%, and an F1 score of 86.2%. These values were superior to those of the clinical model (sensitivity: 70.00%, F1 score: 71.8%) and the radiomics model (sensitivity: 81.67%, F1 score: 83.7%). The radiomics model achieved the highest specificity (87.88%) and PPV (85.96%) in the training set.

In the validation set, the comprehensive model maintained strong generalization ability, achieving the highest sensitivity (96.15%), NPV (95.65%), accuracy (87.04%), and F1 score (87.7%). Although the clinical model also showed relatively high sensitivity (92.31%), its overall balance of performance was inferior to that of the comprehensive model.

The area under the curve (AUC) further supported these findings. In the training and validation sets, the AUCs for the clinical model were 0.7975 (95% CI: 0.7213–0.8736) and 0.8462 (95% CI: 0.7412–0.9511), respectively; for the radiomics model, they were 0.8896 (95% CI: 0.8329–0.9464) and 0.8901 (95% CI: 0.8040–0.9763); and for the comprehensive model, the highest AUCs were obtained as 0.9186 (95% CI: 0.8714–0.9658) and 0.9396 (95% CI: 0.8799–0.9992), indicating superior discriminative capability across both groups.

The optimal classification thresholds for each model, determined by maximizing the Youden index, were 0.5428 for the clinical model, 0.5630 for the radiomics model, and 0.4335 for the comprehensive model. The lower threshold in the comprehensive model reflects its tendency to favor sensitivity, aligning with its enhanced ability to correctly identify high-risk patients. Collectively, these findings demonstrate that the integration of clinical and radiomics features yields more accurate, robust, and clinically useful predictions of pneumothorax risk following microwave ablation.

**Figure 7:**
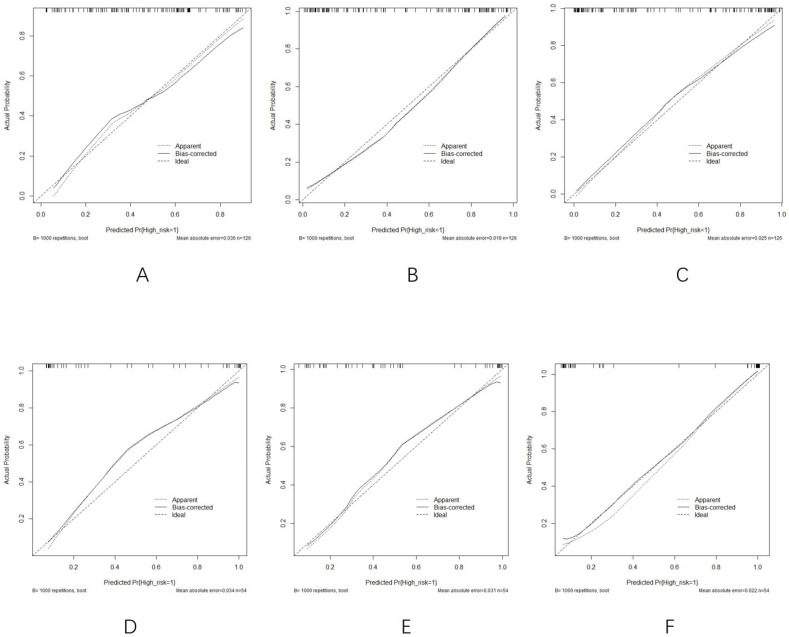
(**A**–**C**) Calibration curves of the (**A**) clinical model, (**B**) radiomics model, and (**C**) comprehensive model in the training group. (**D**–**F**) Calibration curves of the (**D**) clinical model, (**E**) radiomics model, and (**F**) comprehensive model in the validation cohort.

Although there were slight discrepancies between the constructed and predicted values, the model fitting remained consistent. The calibration curves (Figure 7A–F) comparing predicted versus actual probabilities for each model indicated good reliability and consistency among the clinical, radiomics, and comprehensive models.

Overall, the comprehensive model demonstrated superior predictive performance and clinical utility, underscoring its potential as a reliable preoperative tool for identifying high-risk micropapillary and solid LUAD patterns.

## 4. Discussion

This study successfully developed and validated three predictive models—clinical, radiomics, and comprehensive—to preoperatively identify a high-risk MP or SP in LUAD. Our findings demonstrated that integrating radiomics features with conventional clinical and imaging characteristics significantly improves diagnostic accuracy, offering a reliable tool for risk stratification and clinical decision-making. These results are consistent with the conclusions of Wang et al. [27].

### 4.1. Key Findings and Interpretation

The clinical model, based on CT morphological features such as lobulation, spiculation, pleural indentation, and vascular abnormalities, achieved moderate diagnostic performance (AUC: 0.7975–0.8462). These findings are consistent with previous studies reporting associations between an aggressive LUAD pattern and irregular CT features [28,29,30,31]. Interestingly, traditional demographic factors such as age, sex, and smoking history were not significant predictors in our study, likely reflecting the increasing prevalence of LUAD among non-smoking females [32,33].

The radiomics model outperformed the clinical model (AUC: 0.8896–0.8901), underscoring the strength of radiomics features in capturing subtle variations in tumor heterogeneity. The LASSO-selected features—median intensity, skewness, and clustershade—reflect intensity distribution and texture uniformity, which may correlate with the histopathological complexity of MP/SP components [21,34].

The comprehensive model, which integrated clinical features and the RadScore, demonstrated the highest diagnostic efficacy (AUC: 0.9186–0.9396). This model consistently outperformed individual models in both the training and validation cohorts, highlighting the synergistic value of multimodal data integration. The nomogram derived from the ComScore facilitates individualized risk prediction and enhances clinical applicability.

### 4.2. Clinical Implications of the Regression Model

Among all of the predictors, the RadScore was the most significant factor (OR = 5.680, *p* < 0.001), indicating that radiomics biomarkers strongly contribute to risk differentiation. Pleural indentation was the only clinical factor with statistical significance in the final regression model (OR = 3.244, *p* = 0.037), reaffirming its known association with tumor invasiveness [35,36]. While lobulation and spiculation showed borderline significance (*p* = 0.068 and 0.104, respectively), they contributed meaningfully when combined with other features.

To further demonstrate the model’s clinical applicability and support the robustness of our findings, we present the following representative case. A 62-year-old male, a non-smoker with no significant comorbidities, was incidentally found to have a 22 mm solitary pulmonary nodule in the right lower lobe during a routine health examination. The patient was asymptomatic, and physical examination was unremarkable. Contrast-enhanced CT imaging revealed characteristic signs including pleural indentation, vascular convergence, and mild lobulation, raising suspicion of a high-risk histologic subtype. The radiomics analysis generated a high RadScore, further indicating an increased malignancy risk.

Given these findings, a multidisciplinary team recommended anatomic lobectomy over sublobar resection, prioritizing oncologic control based on the predicted histological aggressiveness. The surgical specimen was sent for histopathological evaluation, which confirmed invasive lung adenocarcinoma with predominant micropapillary and solid components. Postoperatively, the patient recovered uneventfully and was referred for adjuvant chemotherapy. This case highlights the utility of integrating radiomics and clinical features to guide personalized surgical planning and therapeutic decision-making, particularly in patients with inconclusive preoperative biopsy results or imaging features suggestive of aggressive tumor subtypes.

### 4.3. Clinical and Therapeutic Relevance

Accurate identification of MP/SP components has direct implications for the following:

**Surgical Planning:** MPs/SPs are associated with higher recurrence rates post-resection [36,37]. The ComScore model supports the selection of lobectomy over sublobar resection for high-risk patients.

**Adjuvant Therapy Decision-Making:** Patients with MP/SP components are more likely to benefit from adjuvant chemotherapy [30]. Preoperative prediction enables early therapeutic planning.

**Minimally Invasive Diagnosis:** Compared with biopsy, ComScore offers a non-invasive, image-based alternative that is particularly useful for nodules unsuitable for pathological sampling.

### 4.4. Comparison with Existing Models

1. Superior Predictive Accuracy and Feature Integration

Compared to the results of prior studies—such as Xing et al.’s biomarker–radiomics model (AUC = 0.894) [37] and Li et al.’s clinical–serum model (AUC = 0.771) [38]—our comprehensive model demonstrated superior performance and broader applicability. Three key advantages are as follows:

**Multimodal Integration:** By combining CT morphological and radiomics features, our model overcomes the limitations of single-modality approaches. Even features with marginal individual significance (e.g., serum markers) can enhance performance when integrated [37].

**Clinical Accessibility:** The nomogram provides an intuitive, software-independent visualization of risk, making the model practical for clinical use.

**Robust Generalizability:** The model maintained high and consistent AUCs in both the training and validation cohorts, indicating strong reproducibility and the potential for widespread adoption.

2. Radiomics Feature Interpretation and Biological Rationale

Although radiomics models have been criticized for their lack of biological interpretability, efforts to relate imaging features to underlying pathophysiology have gained traction in recent years [39,40]. In our study, three first-order radiomics features—median, skewness, and clustershade—were selected via LASSO regression and incorporated into the final model. These features may carry plausible biological meaning:

The Median reflects the central tendency of voxel intensities within a lesion and may relate to the overall cellularity and stromal composition of the tumor. A higher median value might indicate denser fibrotic tissue or compact tumor cell architecture, both of which have been observed in aggressive subtypes of lung cancer [41,42].

Skewness quantifies the asymmetry of intensity distribution, has been associated with intratumoral heterogeneity, necrosis, and hemorrhagic components [40]. In the context of NSCLC, skewness has shown potential correlations with the tumor grade and the extent of necrotic burden [41,42].

Clustershade, a second-order texture feature derived from the gray-level co-occurrence matrix (GLCM), measures the degree of spatial asymmetry and intensity of non-uniformity. Elevated clustershade values may indicate complex architectural patterns, such as irregular glandular structures or infiltrative growth, which are often seen in high-grade malignancies [39,41].

While these interpretations are primarily inferential and based on the existing literature, they enhance the model’s transparency and clinical acceptability. Future studies integrating histopathology or genomic data (radiogenomics) are warranted to further validate the biological significance of these features and facilitate translation into clinical decision-making.

### 4.5. Limitations and Future Directions

Despite the promising results, this study has several limitations:

**Single-Center Design:** This study was conducted using data from a single institution, which may limit the generalizability of the findings due to potential selection bias and institutional variability. To enhance the robustness and external validity of the model, future studies should include prospective validation using multi-center datasets.

**Manual Segmentation:** Manual delineation of tumor regions introduces inter-observer variability, compromising reproducibility and clinical applicability. Future studies should explore automated or deep-learning-based segmentation approaches to enhance robustness.

**Exclusion of Complex or Multifocal Lesions:** This study primarily focused on solitary pulmonary nodules, with complex morphological patterns and multifocal lesions being excluded from the analysis. While this approach enhances the internal consistency and model reliability, it limits the applicability of the findings in real-world clinical settings involving patients with multifocal or irregularly shaped lesions. Future studies should aim to include more diverse lesion types—particularly multifocal or structurally complex nodules—to better assess the model’s generalizability and clinical robustness across broader patient populations.

**Figure 8:**
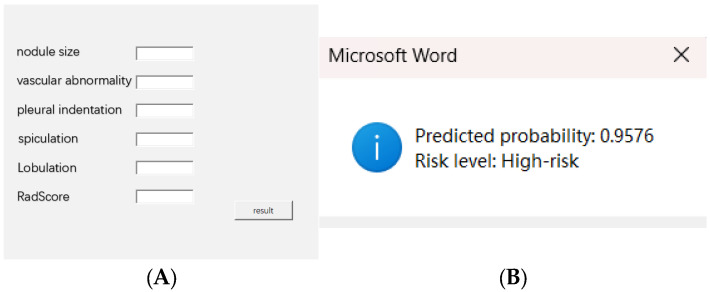
(**A**) Input interface of the Word macro tool with six clinical–radiomics variables. (**B**) Output showing predicted probability and risk classification. The macro tool is available in Supplementary Material Figure S1.

To facilitate clinical use, we developed a Word-based macro tool that enables users to input six key variables and instantly receive a predicted probability and risk classification based on the optimal cutoff. This tool is designed to be lightweight, easily deployable, and operable without requiring advanced software systems. The interface and output demonstration are shown in Figure 8, and the macro-enabled Word file has been provided in Supplementary Material Figure S1 for reference and testing purposes.

Future studies will focus on embedding the model within hospital information systems to enable the automated identification of high-risk patients based on real-time clinical and imaging data, thereby enhancing decision-making efficiency in routine care settings.

## 5. Conclusions

The proposed clinical–radiomics model (ComScore) demonstrates excellent performance in preoperatively identifying a high-risk MP/SP in LUAD. By quantifying individualized risk through an interpretable regression formula and nomogram, this tool facilitates precision-medicine approaches in surgical planning and adjuvant treatment decision-making. Future studies should focus on model automation, external validation, and integration into clinical workflows for optimal utility.

## Figures and Tables

**Table 1:** Clinical baseline data and imaging features of patients.

	Training Group (n = 126)			Validation Group (n = 54)		
	High-Risk Group	Low-Risk Group	*p*	High-Risk Group	Low-Risk Group	*p*
	(n = 60)	(n = 66)		(n = 26)	(n = 28)	
**Age**	63.91 ± 8.69	63.65 ± 10.84	0.881	64.00 ± 9.24	54.57 ± 13.91	0.005
**Height**	161.98 (156, 170)	161.77 (158, 166.25)	0.984	165.54 (159.50, 170.50)	164.04 (158.50, 165.00)	0.379
**Weight**	63.48 (54.00, 71.50)	60.55 (53.00, 67.62)	0.171	68.31 (59.50, 76.25)	63.36 (55.00, 74.50)	0.103
**BMI**	24.10 (21.34, 27.51)	23.10 (20.26, 25.13)	0.152	24.87 (22.48, 27.58)	23.44 (21.01, 26.70)	0.151
**Nodule size**	2.29 (1.50, 2.50)	1.87 (1.30, 2.43)	0.01	2.09 (1.25, 3.25)	1.0 (0.63, 1.10)	<0.001
**Gender**	male 26 (43.3%)	Male 20 (30.3%)	0.129	Male 13 (50%)	Male 18 (64.3%)	0.289
	Female 34 (56.7%)	Female 46 (69.7%)		Female 13 (50%)	Female 10 (35.7%)	
**Smoking history**	No 45 (75%)	No 53 (80.3%)	0.475	No 21 (80.8%)	No 23 (82.1%)	1
	Yes 15 (25%)	Yes 13 (19.7%)		Yes 5 (19.2%)	Yes 5 (17.9%)	
**Lobulated** **sign**	No 14 (23.3%)	No 30 (45.5%)	0.009	No 5 (19.2%)	No 13 (46.4%)	0.034
	Yes 46 (76.7%)	Yes 36 (54.5%)		Yes 21 (80.8%)	Yes 15 (53.6%)	
**Spiculated sign**	No 13 (21.7%)	No 30 (45.5%)	0.005	No 7 (26.9%)	No 17 (60.7%)	0.013
	Yes47 (78.3%)	Yes 36 (54.5%)		Yes 19 (73.1%)	Yes 11 (39.3%)	
**Vacuole** **sign**	No 45 (75%)	No 60 (90.9%)	0.017	No 17 (65.4%)	No 26 (89.3%)	0.012
	Yes 15 (25%)	Yes 6 (9.1%)		Yes 9 (34.6%)	Yes 2 (10.7%)	
**Pleural indentation** **sign**	No 15(25%)	No 37 (56.1%)	<0.01	No 13 (50.0%)	No 22 (78.6%)	0.028
	Yes 45 (75%)	Yes 29 (43.9%)		Yes 13 (50.0%)	Yes 6 (21.4%)	
**Bronchial abnormality sign**	No 34 (56.7%)	No 32 (48.5%)	0.358	No 22 (84.6%)	No 24 (85.7%)	0.91
	Yes 26 (43.3%)	Yes 34 (51.5%)		Yes 4 (15.4%)	Yes 4 (14.3%)	
**Vascular** **abnormality sign**	No 20 (33.3%)	No 35 (53.0%)	0.026	No 6 (23.1%)	No 18 (64.3%)	0.002
	Yes 40 (66.7%)	Yes 31 (47.0%)		Yes 20 (76.9%)	Yes 10 (35.7%)	
**Nodule type**			0.569			0.191
**GGO**	5 (8.3%)	9 (13.6%)		3 (11.5%)	7 (25.0%)	
**CTR < 50%**	10 (16.7%)	8 (12.1%)		3 (11.5%)	4 (14.3%)	
**CTR > 50%**	10 (16.7%)	15 (22.7%)		5 (19.3%)	9 (32.1%)	
**Solid**	35 (58.3%)	34 (51.5%)		15 (57.7%)	8 (28.6%)	

## Data Availability

The data presented in this study are available upon request from the corresponding author due to patient privacy protection and institutional regulations. De-identified radiomics features derived from original medical imaging data can be provided upon approval by the Ethics Committee of the First People’s Hospital of Changzhou.

## Supplementary Materials

The following supporting information can be downloaded at: https://www.mdpi.com/article/10.3390/curroncol32060323/s1. Figure S1: User Interface of the Clinical–Radiomics Prediction Tool.

## References

[1] Thai A.A., Solomon B.J., Sequist L.V., Gainor J.F., Heist R.S., Shaw A.T., Riely G.J., Ramalingam S.S., Gray J.E., Hellmann M.D. (2021). Lung cancer. Lancet.

[2] Pei Q., Luo Y., Chen Y., Li J., Yang X., Zhang L., Wang H., Zhou J., Liu Y., Zheng M. (2022). Artificial intelligence in clinical applications for lung cancer. Clin. Chem. Lab. Med..

[3] Wadowska K., Bil-Lula I., Trembecki Ł., Śliwińska-Mossoń M., Milnerowicz H. (2020). Genetic Markers in Lung Cancer Diagnosis. Int. J. Mol. Sci..

[4] Ashrafi A., Akter Z., Modareszadeh P., Harford T.J., Daneshian M., Asghari W., Amini A., Jahanban-Esfahlan R., Zare P. (2022). Therapeutic Resistance in Lung Cancer. Cancers.

[5] Li Y., Yan B., He S. (2023). Advances in lung cancer treatment. Biomed. Pharmacother..

[6] Butnor K.J. (2020). Histologic subtyping of lung adenocarcinoma. Transl. Lung Cancer Res..

[7] Wilson R., Devaraj A. (2017). Radiomics of pulmonary nodules. Transl. Lung Cancer Res..

[8] Wang W., Hu Z., Zhao J., Zhang X., Chen C., Li Q., Wang Y., Liu J., Zhang L., Li X. (2020). Micropapillary component predicts poor prognosis. J. Cardiothorac. Surg..

[9] Avanzo M., Stancanello J., Pirrone G., Sartor G., Drigo A., Bregant C., Mileto M., Guerrisi A., Russo G., Frezza G. (2020). Radiomics and deep learning in lung cancer. Strahlenther. Onkol..

[10] Wang Y., Hu J., Sun Y., Zhang X., Chen H., Li W., Zhou Q., Liu Y., Zhang Z., Wu Y. (2023). Micropapillary/solid component in stage IA adenocarcinoma. Medicine.

[11] Choi S.H., Jeong J.Y., Lee S.Y., Kim H.R., Kim Y.H., Cho B.C., Lee C.G., Kim D.J., Kim J.H., Park C.K. (2021). Minimal solid/micropapillary component. Thorac. Cancer.

[12] Bensussan A.V., Lin J., Guo C., Zhang Y., Wang L., Chen X., Li H., Liu Y., Zhou J., Yang K. (2020). DESI-MSI in lung cancer diagnosis. Clin. Chem..

[13] Tosi D., Nosotti M., Bonitta G., Righi I., Mendogni P., Rosso L., Palleschi A., Ruggeri P., Santambrogio L., Carrinola R. (2021). Segmentectomy vs. lobectomy. Interact. Cardiovasc. Thorac. Surg..

[14] Zuo S., Wei M., Wang S., Dong D., Tian J., Li X., Liu L., Zheng X., Chen Y., Fang M. (2020). Immune-Cell Characteristic Score in LUAD. Front. Immunol..

[15] Wu Z., Wang F., Cao W., Xu J., Zhang L., Chen R., Li X., Liu Y., Zhou J., Yang X. (2022). Lung cancer risk prediction models. Thorac. Cancer.

[16] Kim Y.J., Lee H.J., Kim K.G., Lee S.H., Kim Y.H., Han D.H., Kim H.J., Choi Y.H., Park C.M. (2019). CT parameters and radiomic features. Comput. Math. Methods Med..

[17] Kumar V., Gu Y., Basu S., Berglund A., Eschrich S.A., Schabath M.B., Forster K., Aerts H.J., Dekker A., Fenstermacher D. (2012). Radiomics: Process and challenges. Magn. Reson. Imaging.

[18] Hatt M., Krizsan A.K., Rahmim A., Bradshaw T.J., Costa P.F., Forgacs A., Seifert R., Zwanenburg A., El Naqa I., Kinahan P.E. (2023). Radiomics guideline. Eur. J. Nucl. Med. Mol. Imaging.

[19] Mayerhoefer M.E., Materka A., Langs G., Häggström I., Szczypiński P., Gibbs P., Cook G. (2020). Introduction to Radiomics. J. Nucl. Med..

[20] Choi E.R., Lee H.Y., Jeong J.Y., Choi Y.L., Kim J.H., Kim H.K., Shim Y.M., Lee K.S. (2016). Quantitative image variables in LUAD. Oncotarget.

[21] Sala E., Mema E., Himoto Y., Veeraraghavan H., Mikheev A., Yoshida H., Vargas H.A., Galluzzo A., Goldman D.A., Jambawalikar S. (2017). Radiogenomics and habitat imaging. Clin. Radiol..

[22] Bousabarah K., Temming S., Hoevels M., Koch D., Borggrefe J., Nowak S., Desideri S., Cirillo M., Giraud P., Nierer L. (2019). Radiomic analysis for radiation injury. Strahlenther. Onkol..

[23] Liang Z.R., Lv F.J., Fu B.J., Li X.T., Wang W., Dong D., Tian J., Fang M.J., Liu L., Zheng X.C. (2023). Reticulation Sign on Thin-Section CT: Utility for Predicting Invasiveness of Pure Ground-Glass Nodules. Am. J. Roentgenol..

[24] Song L., Xing T., Zhu Z., Yang F., Li J., Wang J., Liu J., Zhang J., Zhang Y., Xie Y. (2021). Hybrid Clinical-Radiomics Model for Precisely Predicting the Invasiveness of Lung Adenocarcinoma Manifesting as Pure Ground-Glass Nodule. Acad. Radiol..

[25] Yagi T., Yamazaki M., Ohashi R., Ogura T., Masuda M., Matsumoto Y., Hattori A., Matsuoka T., Nagashima T., Tomizawa K. (2018). HRCT texture analysis for pure or part-solid ground-glass nodules: Distinguishability of adenocarcinoma in situ or minimally invasive adenocarcinoma from invasive adenocarcinoma. Jpn. J. Radiol..

[26] Wang J., Ma H., Ni C.J., Zhang L., Wang Y., Liu Y., Chen X., Li W., Zhou Q., Sun Y. (2019). Clinical characteristics and prognosis of ground-glass opacity nodules in young patients. J. Thorac. Dis..

[27] Wang Z., Zhang N., Liu J., Liu J. (2023). Predicting Micropapillary or Solid Pattern of Lung Adenocarcinoma with CT-Based Radiomics, Conventional Radiographic, and Clinical Features. Respir. Res..

[28] Zhang Y.P., Heuvelmans M.A., Zhang H., Oudkerk M., Vliegenthart R., Xie X.Q. (2018). Changes in quantitative CT image features of ground-glass nodules in differentiating invasive pulmonary adenocarcinoma from benign and in situ lesions: Histopathological comparisons. Clin. Radiol..

[29] Miao Y., Zhang J., Zou J., Zhu Z., Li W., Zhou Q., Chen H., Liu Y., Wang Y., Sun Y. (2017). Correlation in histological pattern with high resolution computed tomography signatures of early-stage lung adenocarcinoma. Transl. Lung Cancer Res..

[30] Gao F., Sun Y., Zhang G., Li Z., Wang H., Chen X., Liu Y., Zheng M., Yang X., Li J. (2017). CT characterization of different pathological types of subcentimeter pulmonary ground-glass nodular lesions. Br. J. Radiol..

[31] Domagala-Kulawik J., Trojnar A., Safianowska A., Nowicka U., Walkiewicz D., Maskey-Warzechowska M., Chazan R. (2020). Lung cancer in women in 21th century. J. Thorac. Dis..

[32] Lau S.C.M., Pan Y., Velcheti V., Wong K.K., Sacher A.G., Costa D.B., Neal J.W., Sholl L.M., Heist R.S., Wirth L.J. (2022). Squamous cell lung cancer: Current landscape and future therapeutic options. Cancer Cell.

[33] Holzinger A., Haibe-Kains B., Jurisica I. (2019). Why imaging data alone is not enough: AI-based integration of imaging, omics, and clinical data. Eur. J. Nucl. Med. Mol. Imaging.

[34] Sata Y., Nakajima T., Fukuyo M., Morimoto J., Hara T., Matsuura R., Ihara S., Miyashita Y., Horie M., Matsusaka K. (2020). High expression of CXCL14 is a biomarker of lung adenocarcinoma with micropapillary pattern. Cancer Sci..

[35] Matsushima K., Sonoda D., Mitsui A., Kitazono I., Ikeda R., Yoshizawa A., Tsuchiya T., Nagayasu T. (2022). Factors associated with lymph node metastasis upstage after resection for patients with micropapillary lung adenocarcinoma. Thorac. Cancer.

[36] Wang C., Shao J., Lv J., Deng L., Xu H., Wang Z., Zhang Y., Liu Y., Zheng M., Yang X. (2021). Deep learning for predicting subtype classification and survival of lung adenocarcinoma on computed tomography. Transl. Oncol..

[37] Xing X., Li L., Sun M., Zhang Y., Wang Z., Liu Y., Chen X., Li W., Zhou Q., Sun Y. (2024). A combination of radiomic features, clinic characteristics, and serum tumor biomarkers to predict the possibility of the micropapillary/solid component of lung adenocarcinoma. Ther. Adv. Respir. Dis..

[38] Li Z., Wu W., Pan X., Chen H., Liu Y., Zhang Z., Wang Y., Sun Y., Zhou Q., Li W. (2022). Serum tumor markers level and their predictive values for solid and micropapillary components in lung adenocarcinoma. Cancer Med..

[39] Yip S.S.F., Aerts H.J.W.L. (2016). Applications and limitations of radiomics. Phys. Med. Biol..

[40] Ganeshan B., Miles K.A., Young R.C.D., Chatwin C.R. (2013). Quantifying tumour heterogeneity with CT. Cancer Imaging.

[41] Lubner M.G., Smith A.D., Sandrasegaran K., Sahani D.V., Pickhardt P.J. (2017). CT Texture Analysis: Definitions, Applications, and Limitations. Radiographics.

[42] Kirienko M., Cozzi L., Antunovic L., Lozza L., Fogliata A., Voulaz E., Rossi A., Chiti A., Sollini M. (2018). Prediction of disease-free survival by PET/CT radiomic signature in NSCLC. Eur. J. Nucl. Med. Mol. Imaging.

