# Supplementary material for: A Clinical–Radiomics Nomogram for the Preoperative Prediction of Aggressive Micropapillary and a Solid Pattern in Lung Adenocarcinoma
Source: Curr Oncol. 2025 May 30;32(6):323. doi: 10.3390/curroncol32060323 (PMC12192257; doi:10.3390/curroncol32060323)
Supplement: Supplementary file 1 [file curroncol-32-00323-s001.zip › curroncol-3613472-supplementary.pdf]

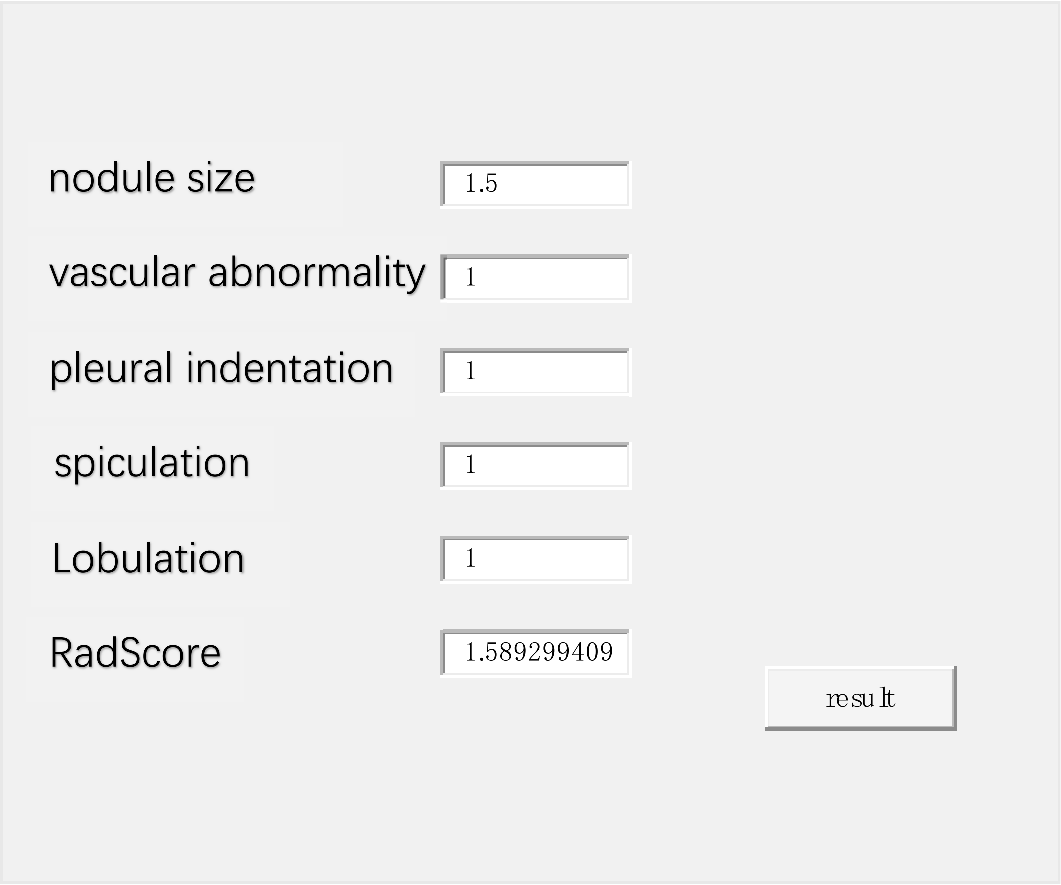
nodule size
1.5
vascular abnormality
1
pleural indentation
1
spiculation
1
Lobulation
1
RadScore
1.589299409
result
